# Rational engineering of *Escherichia coli* strain for stable and enhanced biosynthesis of pinene

**DOI:** 10.3389/fmicb.2024.1527113

**Published:** 2025-01-07

**Authors:** Muhammad Hammad Hussain, Lu Han, Yanlong Wei, Muhammad Javid, Kamran Ashraf, Maria Martuscelli, Waleed Aldahmash, Meijin Guo, Ali Mohsin, Zhanxia Li

**Affiliations:** ^1^State Key Laboratory of Bioreactor Engineering, East China University of Science and Technology, Shanghai, China; ^2^Department of Microbiology, Faculty of Chemical and Life Sciences, Abdul Wali Khan University Mardan, Mardan, Pakistan; ^3^Department of Bioscience and Food, Agricultural and Environmental Technology, University of the Studies of Teramo, Teramo, Italy; ^4^Zoology Department, College of Science, King Saud University, Riyadh, Saudi Arabia; ^5^Department of Pulmonary and Critical Care Medicine, Shanghai Sixth People’s Hospital Affiliated to Shanghai Jiao Tong University School of Medicine, Shanghai, China

**Keywords:** biofuel, chromosomal integration, rational design model, pinene, fermentation, Escherichia coli, batch fermenter

## Abstract

Monoterpene *α*-pinene exhibits significant potential as an alternative fuel, widely recognized for its affordability and eco-friendly nature. It demonstrates multiple biological activities and has a wide range of applications. However, the limited supply of pinene extracted from plants poses a challenge in meeting the needs of the aviation industry and other sectors. Considering this, the microbial cell factory is the only viable option for achieving sustainable pinene production. This study employed a rational design model to optimize the copy number and integration site for the heterogenous pinene expression pathway in *Escherichia coli* (*E. coli*). The integrated strain with the best pinene pathway PG1 was selected. Subsequently, the resulting strain, *E. coli* HSY009, accumulated 49.01 mg/L of pinene after 24 h fermentation in the flask culture. To further enhance production, pinene expression cassette PG1 was sequentially integrated into three non-essential regions (44th, 58th, 23rd), resulting in an improved pinene titer. Then, the fermentation process under optimized conditions enhanced the production of pinene to 436.68 mg/L in a 5 L batch fermenter with a mean productivity of 14.55 mg/L/h. To the best of our knowledge, this work represents the maximum mean pinene productivity based on the currently available literature. The findings of this work provide valuable insights for optimizing *E. coli* to produce other valuable terpenoids that share the same intermediates, IPP and DMAPP. Conclusively, this research validates the model’s universality and highlights its potential for application as cutting-edge biofuel precursors.

## 1 Introduction

There is a widely reported decrease in fossil fuel levels and a continuous increase in energy requirements, particularly in developing countries (Hussain, 2024). To address these issues, the generation of substitute fuels has gained significant attention. Replacement of traditional fossil fuels with substitutes can substantially reduce greenhouse gasses in the environment (Liu et al., 2023). Thus, the search for economic and alternative energy sources has become paramount. In this respect, biofuels derived from microbial sources are being extensively investigated. These microbial fuels offer a beneficial alternative energy option that can supplement or even replace conventional fuels like jet fuel or diesel without requiring infrastructure advancements or engine adjustments (Carruthers et al., 2023). Recently, pinene dimer has been characterized for its high energy density, comparable to JP-10 fuel (Bierkandt et al., 2020). Due to its high functionality, pinene dimer is considered an important commodity and can be produced by chemical dimerization of pinene, thereby contributing to increased fuel density (Bierkandt et al., 2020).

In nature, *α*-pinene exists as an active monoterpene and has gained increasing popularity in various industries, including food, cosmetic, therapeutic, and nutraceuticals (Salehi et al., 2019). Therefore, its production is experiencing a growing trend, and the market for *α*-pinene is projected to expand at an annual growth rate of 3.2%. By 2023, the estimated market size for pinene is expected to reach the value of 201.1 million USD dollars, with a market price of approximately 2.5 USD dollars/kg (Global Alpha Pinene Market, 2024). Commercially, *α*-pinene is produced in paper mills either as a by-product or obtained by tapping pine trees (Tsolakis et al., 2019). However, the extraction of α-pinene from various plants, including rosemary, sage, wild thyme, and a variety of conifers is found to be inefficient and insufficient to meet the increased demand for commercial applications (Sameer et al., 2012). This limitation may arise due to a limited supply of natural bioactive compounds, high processing costs, and lengthy operational time. At the same time, adopting microbial strategy and its associated benefits such as sustainable production and fast growth are attracting strong interest as highly appealing alternatives. Numerous studies have revealed the existence of two primary pathways, namely the mevalonate (MVA) pathway and the methylerythritol 4-phosphate (MEP) pathway. These pathways improve the supply of precursors: isopentenyl diphosphate (IPP) and dimethylallyl diphosphate (DMAPP) for terpenoid production, particularly pinene and lycopene in engineered microbial strains (Rinaldi et al., 2022; Hussain et al., 2024). Even though a variety of microorganisms are known to produce intermediates DMAPP and IPP through MVA or MEP pathways, they cannot synthesize pinene due to the absence of pinene synthase. This challenge can be addressed by introducing the desired gene in the target host either by plasmid-based methods for gene overexpression or by integrating the gene into the host chromosome via CRISPR-associated 9 (CRISPR/Cas9) and *λ*-Red recombination system (Gu et al., 2015). Furthermore, the better selection of hosts and optimization of heterogenous pathways are crucial prerequisites for the enhanced production of biofuels (Okoro et al., 2022). In this regard, the *Saccharomyces*, *Bacillus*, and *Cyanobacteria* species are well-acknowledged pinene-producing microorganisms in terms of their ability to utilize both recombinant MEP or MVA pathway or express isoprene synthase (Kim et al., 2016; Zhao et al., 2011). Particularly, the ability of *E. coli* to grow on several types of chemically defined medium and its extensive genomic data makes it the most suitable option for synthesizing pinene (Zhao et al., 2011).

Genome engineering techniques, including CRISPR/Cas9 and lambda Red, have significantly enhanced the efficiency, speed, and accuracy of generating modified bacteria for biofuel production. For example, the level of 2-Phenyl ethanol was increased in *Kluyveromyces marxianus* by inserting a multigene cassette through CRISPR-Cas9 (Li et al., 2021). Furthermore, Liu et al. (2017) successfully applied the CRISPR/Cas9 system for the genomic engineering of four gene loci in *Myceliophthora thermophila* for desired industrial outcomes (Liu et al., 2017). Another study integrated a tunable intergenic region between the *Pinus taeda* *Pt1*^Q457L^ and *A. grandis GPPS*^D90G/L175P^ of *E. coli* strain TZFP through the CRISPR/Cas9 system. This integration regulates multiple gene expressions and creates genomic balance. The resulting engineered microbial *consortia* (*E. coli-E. coli*) generated 166.5 mg/L pinene (Niu et al., 2018). Similarly, Huang et al. (2022) reported the optimal pinene production of 14.3 mg/L in *E. coli* (*MG1655BFN*) by using an I-SceI cutting system that replaced the promoter with P37 to regulate the expression of *ndk*, *acrB*, and *flgFG* genes (Huang et al., 2022).

In the present work, the primary focus was on developing the integrated strain through CRISPR/Cas9 and lambda red recombineering to achieve optimal biofuel production, with a particular emphasis on the production of pinene. The starting strain used in this research was our previously engineered lycopene-producing strain, DH411 (Wei et al., 2018). A copy of the expression element S1 was integrated at the *LpxM* site to construct the pinene-producing strain. An integrated pinene-producing engineered strain *Escherichia coli* was constructed by integrating one copy of expression element PG1 counterclockwise in region 8 and knocking off the lycopene biosynthesis pathway in region 23. Although the resulting strain was successfully modified through the CRISPR/Cas9/lambda-Red techniques, its pinene production was low (49.01 mg/L). The issue of lower production has been resolved by exploiting chromosomal positioning through CRISPR/Cas9 that positively influences gene transcription, further improving the desired bioproduct’s production level (Bryant et al., 2014; Gerganova et al., 2015). Thus, the current study uses CRISPR/Cas9 and lambda-Red systems to establish a rational design model to evaluate the copy number and chromosomal integrated position for optimal results in the host strain. Through optimization efforts, the final HSY012 strain was cultivated in a shake flask, resulting in an increased pinene concentration. Ultimately, in the 5 L bioreactor under optimized conditions, to the best of our knowledge, our rational genetic modification has empowered the engineered *E. coli* to achieve the highest reported mean pinene productivity of 14.55 mg/L/h in batch culture mode to date.

## 2 Results

### 2.1 Construction of integrated engineering strain for pinene production

**Figure 1:**
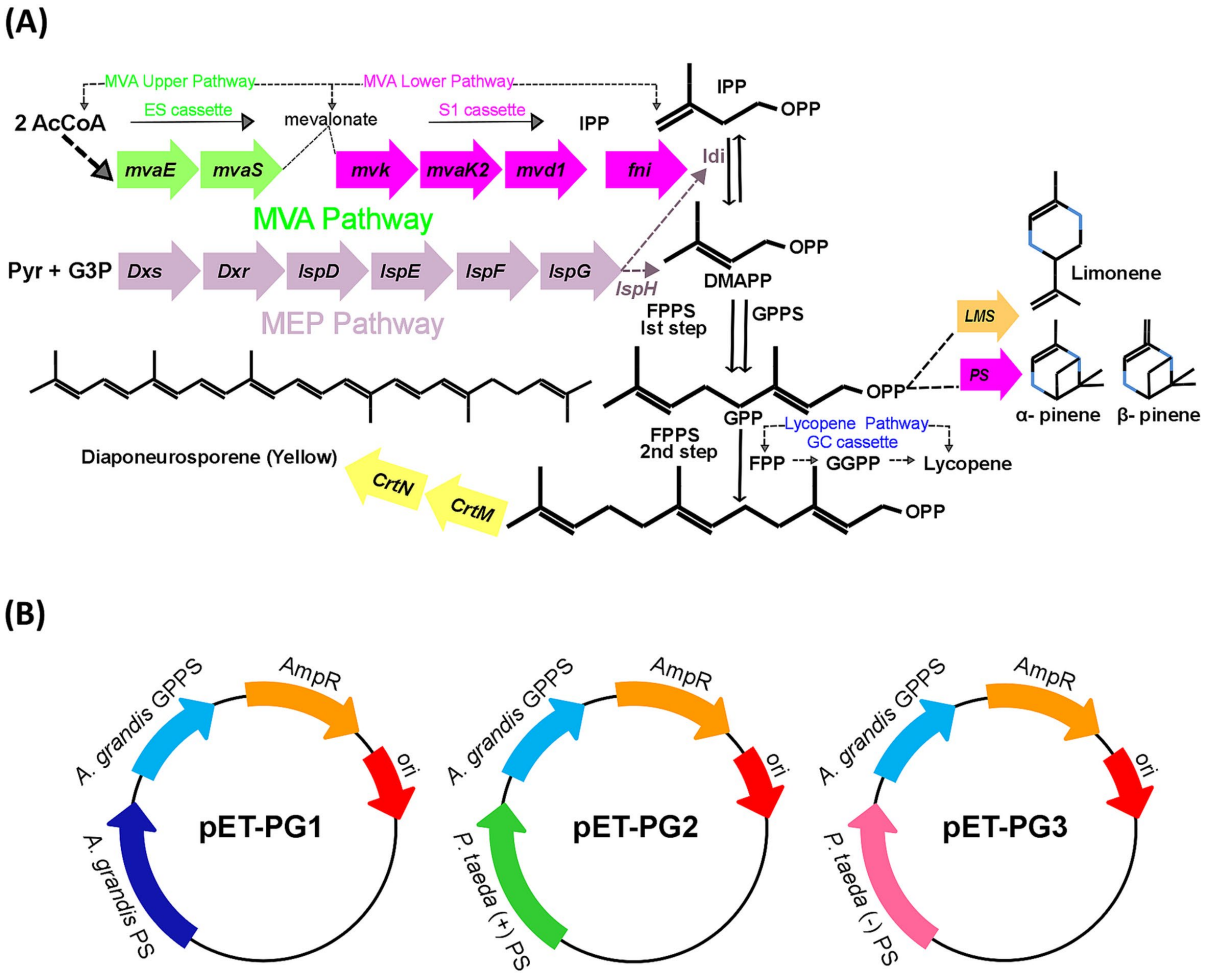
**(A)** Schematic representation of pinene pathway. AcCoA: acetyl-CoA; DMAPP: isomer dimethylallyl diphosphate; IPP: isopentenyl diphosphate; FPP: farnesyl pyrophosphate; GGPP: geranylgeranyl diphosphate; GPP: geranyl diphosphate. **(B)** Graphical representation of bacterial plasmids pETL-PG1, pETL-PG2, and pETL-PG3, also denoted as (pET-PG1, pET-PG2, pET-PG3) used to amplify heterogeneous genes of pinene pathway. GPPS: geranyl diphosphate synthase; PS: pinene synthase.

Genes were simplified as three elements: ES (upper MVA pathway), S1 (lower MVA pathway), and PG (Pinene synthetic pathway; Figure 1A) cassettes to drive heterologous protein expression for improved pinene biosynthesis (Figure 1B).

**Figure 2:**
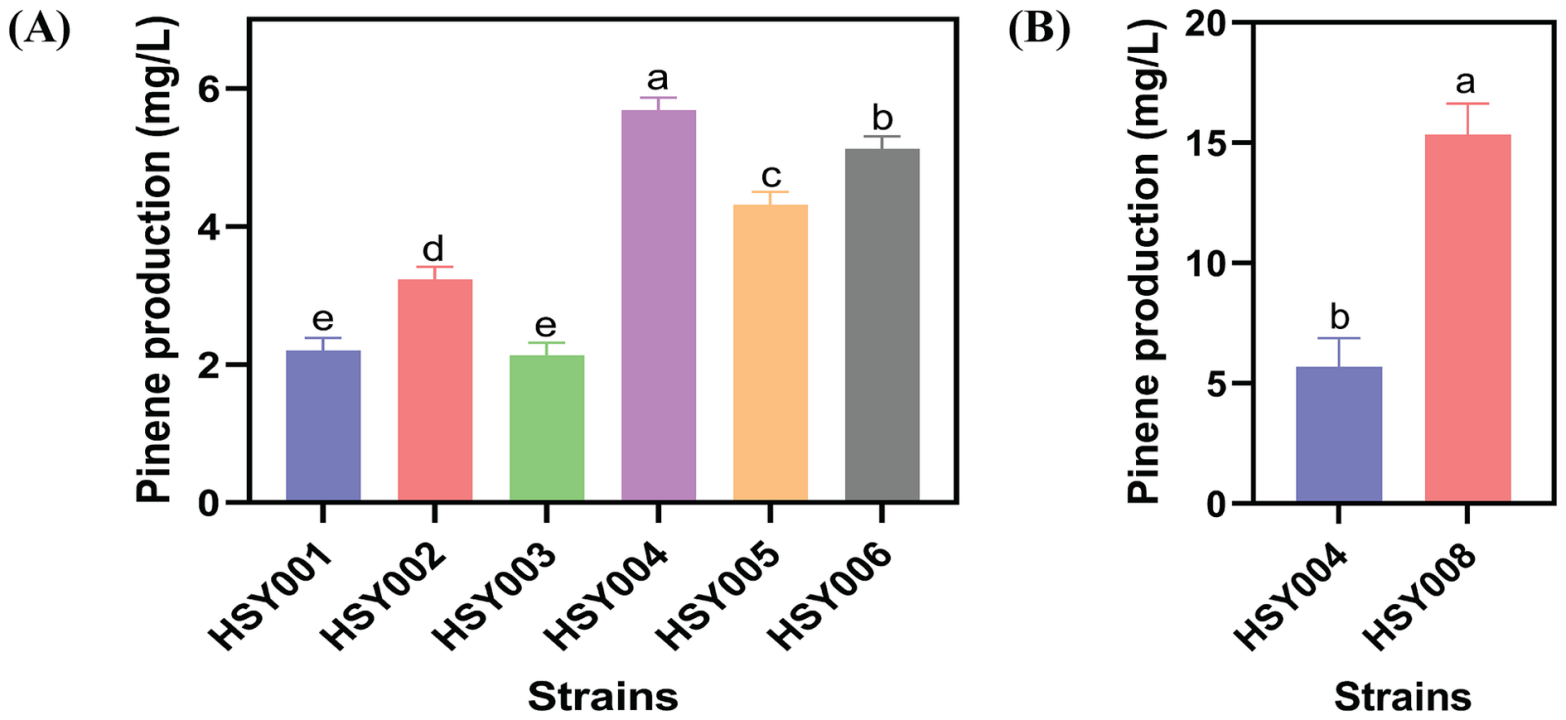
Pinene level of strains. **(A)** Pinene production in six integrated strains: HSY001, HSY002, HSY003, HSY004, HSY005, and HSY006. **(B)** Comparison between the pinene production of HSY004 and HSY008. The controlled experiments were conducted using a 5 mL medium at 220 rpm and 32°C in a test tube. Data are expressed as the mean values ± standard deviation (*n* = 3). Values in columns with different letters are significantly different at a 5% level, according to Tukey’s Highest Difference Test.

Three pinene pathways, PG1, PG2, and PG3, from different species, were constructed (Figure 1B). To verify which heterogeneous expression pathway is needed for optimum pinene production in strains DH411 and DH416. In this regard, the strain DH416 was transformed with plasmid pETL carrying PG1, PG2 and PG3 cassettes to generate three strains HSY001 (DH416/pETL-PG1), HSY002 (DH416/pETL-PG2) and HSY003 (DH416/pETL-PG3), respectively. On the other hand, three more strains HSY004 (DH411/pETL-PG1), HSY005 (DH411/pETL-PG2), HSY006 (DH411/pETL-PG3) were generated. Lycopene and pinene are classified as terpenoids and share similar precursor requirements (IPP and DMAPP). When the activity of the pinene pathway was increased, the intracellular precursor levels for lycopene biosynthesis were decreased. This occurs because both pinene and lycopene synthesis pathways compete for the same precursors. Strain (DH411/pETL-PG1) with the lowest lycopene level was selected for further experiments, indicating the corresponding pinene synthetic pathway (PG1) is more appropriate than PG2 and PG3 (Supplementary Figure S1). According to the result, it was difficult to transform DH416 into a pinene-producing strain, hence *E. coli* DH411 was selected as the starting strain. For instance, Yang et al. (2013) found that an accumulation of 5.44 mg/L pinene was achieved in *E. coli* via heterologous GPPS (*A. grandis*) and PS (*P. taeda*) expression (Yang et al., 2013). Similar findings have also been reported by Sarria et al. (2014), who demonstrated that the heterologous expression of PS (*A. grandis*) and GPPS (*A. grandis*) generated 28 mg/L of pinene in *E. coli* (Sarria et al., 2014). Afterwards, the pinene production from these six strains was analyzed (Figure 2A).

Based on the above-mentioned results, strain HSY004 (DH411/pETL-PG1) was identified as the most favorable integrated strain in comparison to other strains. It exhibited the maximal level of pinene production about 5.66 mg/L (Figure 2A). The PG1 cassette was determined to be the optimal choice for heterologous pinene production. To further enhance pinene production, integration of one PG1 cassette in a counterclockwise orientation in region 8 was performed, resulting in the creation of strain HSY007. DH411 strains lacked the expression cassette for pinene synthesis at the 8th site. After that, the GC cassette was knocked off in region 23 of strain HSY007 to construct a new strain HSY008. While comparing the pinene production of both HSY004 and HSY008, it was found that the integrated strain HSY008 generated a 3-fold increase (15.34 mg/L) in pinene production when compared to strain HSY004 (Figure 2B). More so, it was found that the knockout of the lycopene biosynthesis pathway (GC) resulted in enhanced pinene production in integrated strain HSY008.

**Figure 3:**
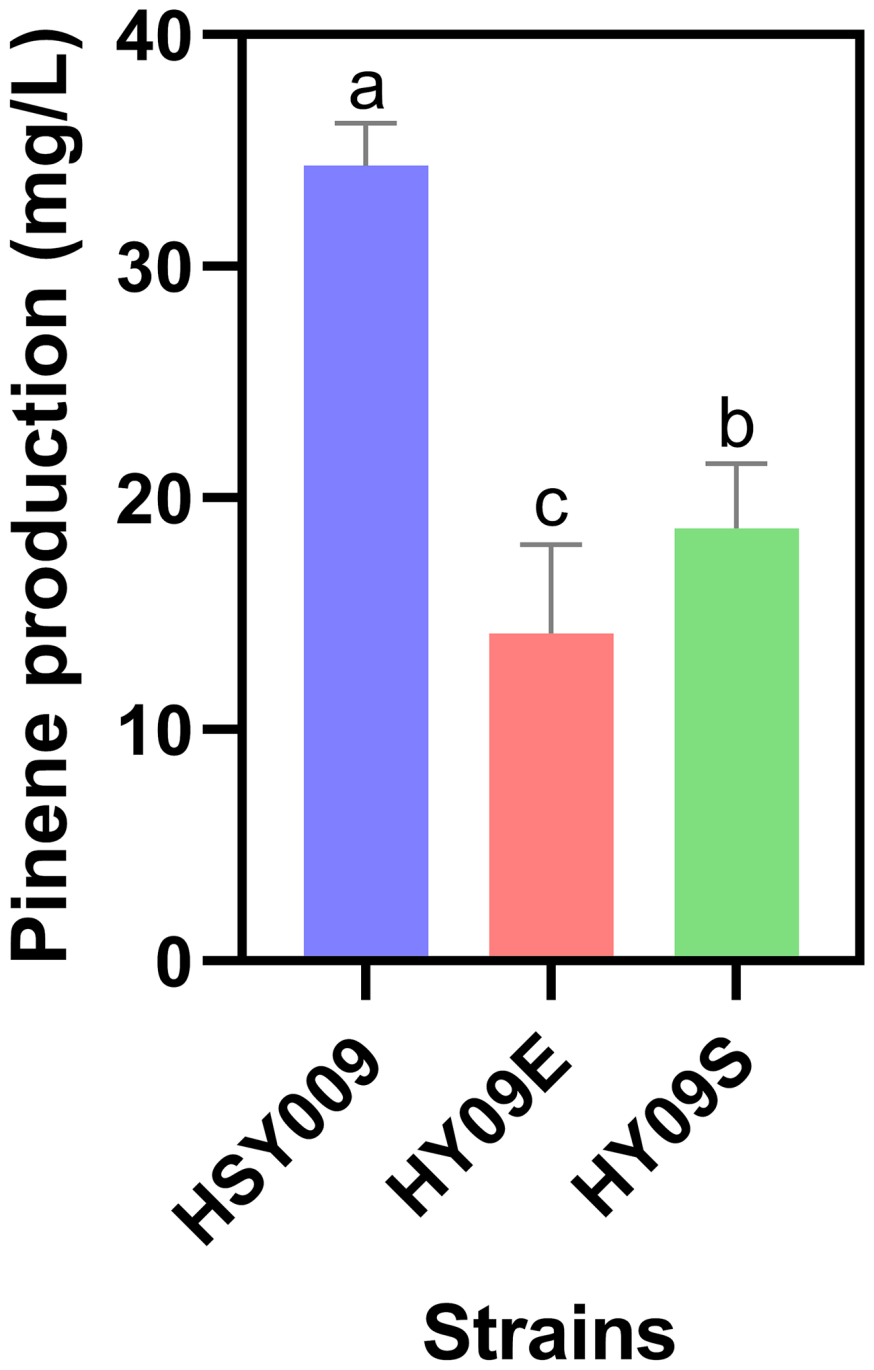
Pinene production in three integrated strains: HSY009, HY09E, and HY09S. The controlled experiments were conducted using a 5 mL medium at 220 rpm and 32°C in a test tube. Data are expressed as the mean values ± standard deviation (*n* = 3). Values in columns with different letters are significantly different at a 5% level, according to Tukey’s Highest Difference Test.

To investigate whether further overexpression of either the upper MVA pathway or lower MVA pathway was necessary, strain HSY009 was generated. In this strain, one S1 cassette was integrated at the *LpxM* region (non-essential position). This integrated strain consisted of two copies of S1 cassette which is more than that of starting strain DH411. Then, the integrated strain HSY009 was transformed with the single copy plasmid pCC1FOS carrying ES and S1 cassettes, which in turn generated the HY09E (HSY009/pCC1E) and HY09S (HSY009/pCC1S) strains. Notably, the increase in either the MVA upper pathway or the MVA lower pathway to generate HY09E (*ES: S1: PG 2*: 2: 1), and HY09S (*ES: S1: PG 1*: 3: 1) strains did not enhance the pinene production, as shown in Figure 3. The pinene production of HSY009 (*ES: S1: PG 1*: 2: 1) was 34.35 mg/L, which is significantly highest among three newly constructed strains HY09E, and HY09S, respectively (Figure 3). Our findings align with the study of Hussain et al. (2021), who stated that a further increase in either the lycopene expression pathway or MVA lower pathway was not beneficial in terms of specific lycopene production after the generation of 11 models (Hussain et al., 2021). This may be due to the phenotype mimicking the loss-of-function mutation when there is a high gene expression level. Hence, the strain HSY009 was declared to be the best-performing strain. This integrated strain was comprised of one copy of the upper MVA pathway, two copies of the lower MVA pathway, and one copy of the pinene synthetic pathway. Therefore, understanding the copy number of heterogeneous expression pathways is crucial for designing an efficient expression pathway.

### 2.2 Effect of integrated position on pinene production

**Figure 4:**
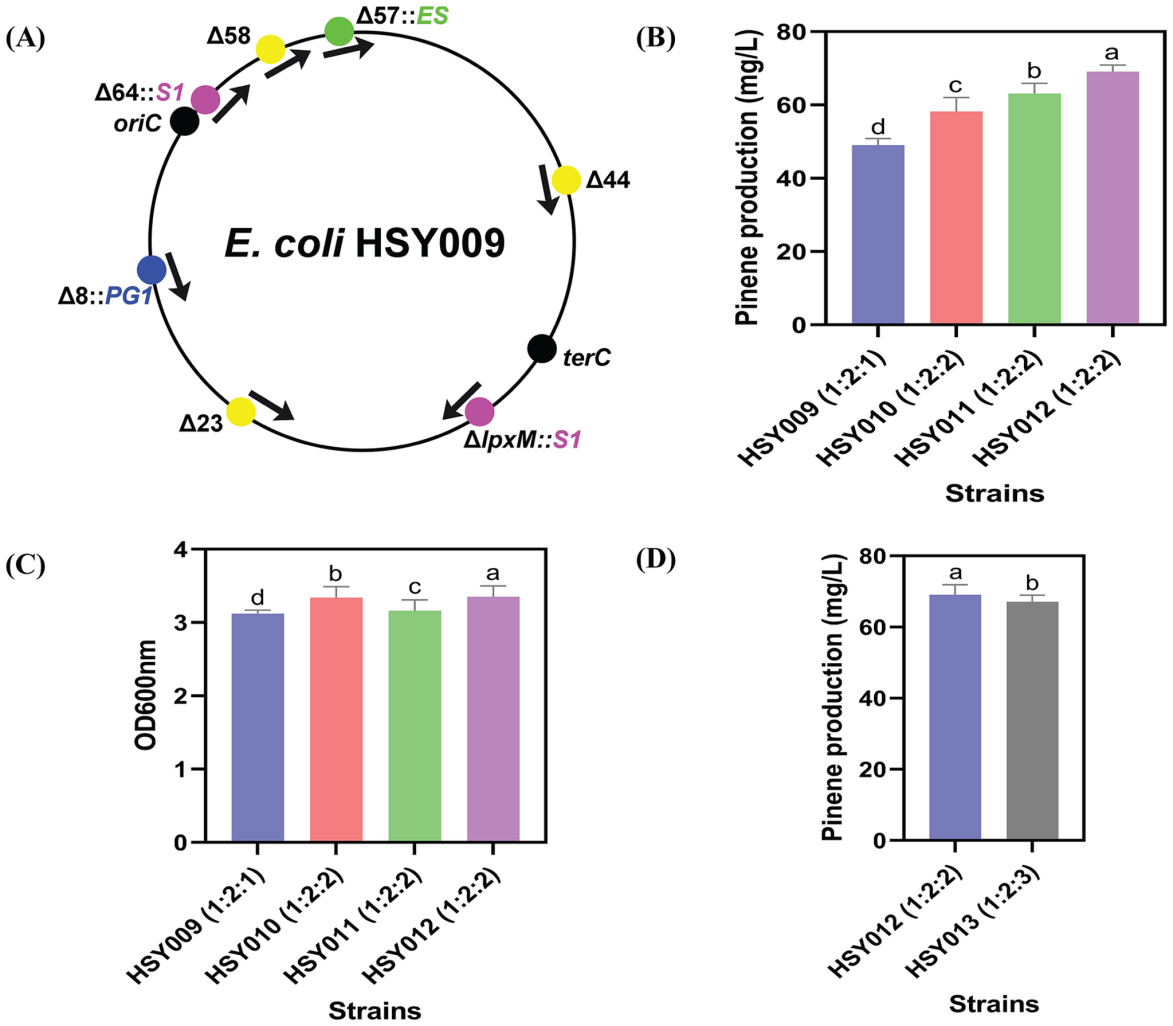
Integrated positions for pinene synthetic pathway, pinene level, and growth ability of strains. **(A)** Three regions (44th, 58th, and 23rd regions) were selected to integrate pinene synthetic pathways. **(B)** A pinene production of four strains: HSY009, HSY010, HSY011, and HSY012. **(C)** Comparison between the growth rate of four integrated strains HSY009, HSY010, HSY011 and HSY012. **(D)** Pinene production of two integrated strains, HSY012 and HSY013. The controlled experiments were conducted using a 5 mL medium at 220 rpm and 32°C in a test tube. Data are expressed as the mean values ± standard deviation (*n* = 3). Values in columns with different letters are significantly different at a 5% level, according to Tukey’s Highest Difference Test.

To improve the pinene production in strain HSY009, the expression cassettes PG were integrated into three non-essential regions of the genome. These regions were the 44th, 58th, and 23rd, as illustrated in Figure 4A. The region’s selection was due to its wide distribution throughout the chromosome (Wei et al., 2017).

By integrating the PG cassettes one by one into these regions, three new strains were generated: HSY010 (HSY009 Δ44th::T7 PG1), HSY011 (HSY009 Δ58th::T7 PG1), and HSY012 (HSY009 Δ23rd::T7 PG1). Moreover, it was found that the best pinene production of 69.08 mg/L belonged to strain HSY012, as depicted in Figure 4B.

These results indicated that the integrated strain HSY012 (*ES: S1: PG* 1: 2: 2) carrying one copy of the MVA upper pathway, two copies of the MVA lower pathway, and two copies of the pinene synthetic pathway, exhibited the highest pinene production followed by HSY011, HSY010, and HSY009 strains, respectively (Figure 4B). Furthermore, it was observed that the growth rate of the HSY012 strain was higher compared to strains HSY011, HSY010, and HSY009, as depicted in Figure 4C. This finding suggests that integrating the PG1 cassette at the 23rd region increased pinene production and growth rate. This result is in line with Wei et al. (2017), who compared the lycopene level by changing the direction (clockwise and counter-clockwise direction) and position (8th, 23rd, 58th, and 64th nonessential regions) of GC (Lycopene expression pathway) integration. It was found that the coupling integration positions with expression direction resulted in a 5- and 10-fold increase in lycopene production in *E. coli* (Wei et al., 2017).

**Figure 5:**
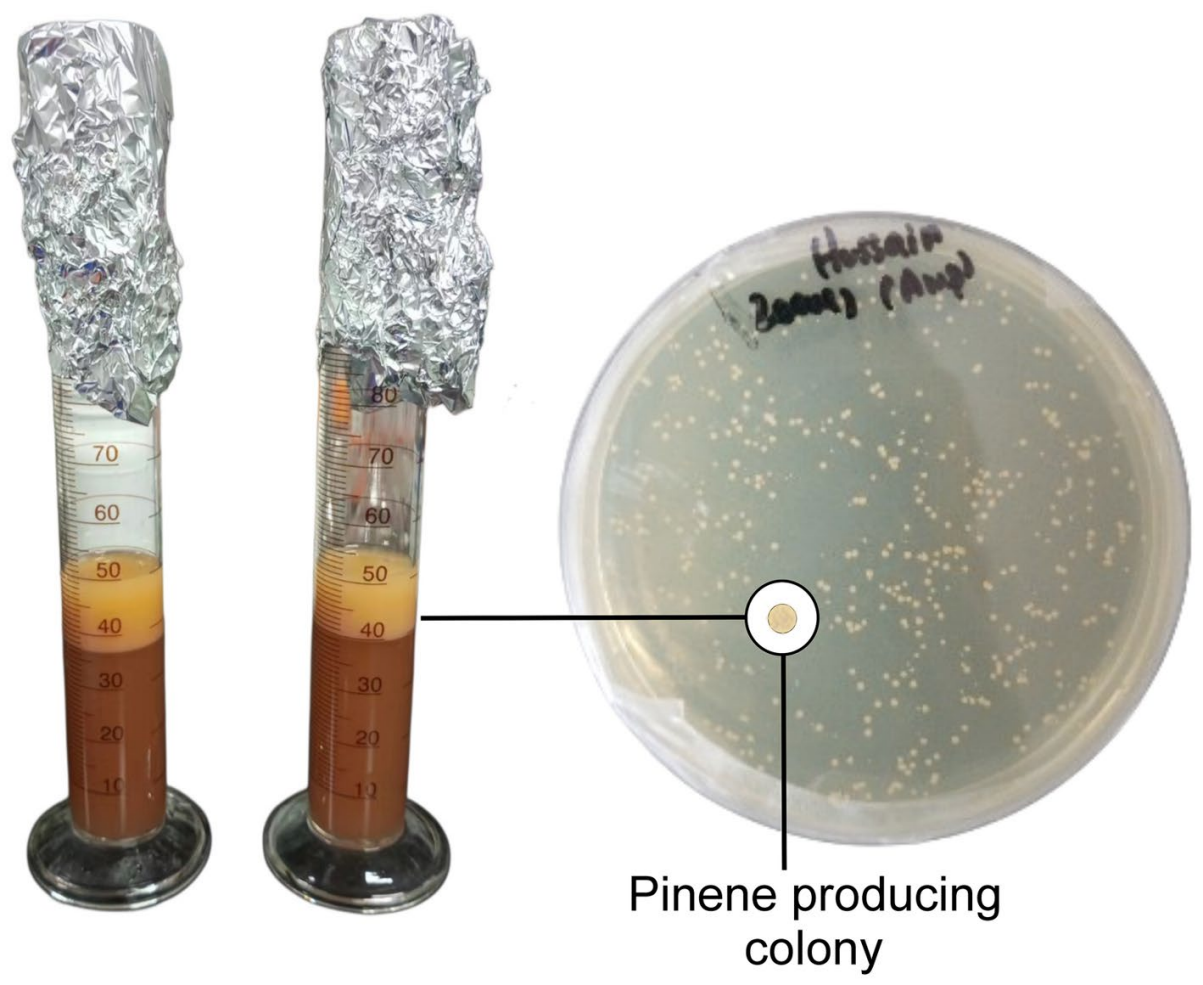
HSY012 colonies on an agar plate and its pinene production.

To test whether the overexpression of the pinene expression pathway was required after the development of HSY012, the transformation of a single copy plasmid pCC1FOS carrying PG1 cassette was executed in strain HSY012. This transformation resulted in the generation of HSY013 (HSY012/pCC1P) strain. However, there was no significant increase in the pinene yield, as shown in Figure 4D. This result indicated that the overexpression of the pinene synthetic pathway was futile at this point, as the HSY012 strain (*ES: S1: PG* 1: 2: 2) accumulated more pinene up to 69.08 mg/L than that of HSY013 strain (*ES: S1: PG* 1: 2: 3) (Figure 4D). Therefore, HSY012 was identified as the best-performing strain among the tested strains. It consisted of one copy of the upper MVA pathway, two copies of the lower MVA pathway, and two copies of the pinene synthetic pathway. Table 1 summarizes the pinene level (mg/L) of five recombinant *E. coli* strains after integrating the pinene expression pathway at different integration sites. The colony formation and pinene production are illustrated in Figure 5.

**Table 1:** Pinene level of recombinant pinene-producing *E. coli* strains.

Strain	HSY009	HSY010	HSY011	HSY012	HSY013
Pinene level (mg/L)	49.01 ± 0.38^e^	58.21 ± 0.34^d^	63.08 ± 0.69^c^	69.08 ± 0.46^a^	66.79 ± 0.85^b^
Integration sites	8	44	58	23	23

Data are presented as the mean values ± standard deviation (*n* = 3). The different letters in the row show that the mean values are significantly different.

### 2.3 Effect of optimized fermentation conditions on pinene production

**Figure 6:**
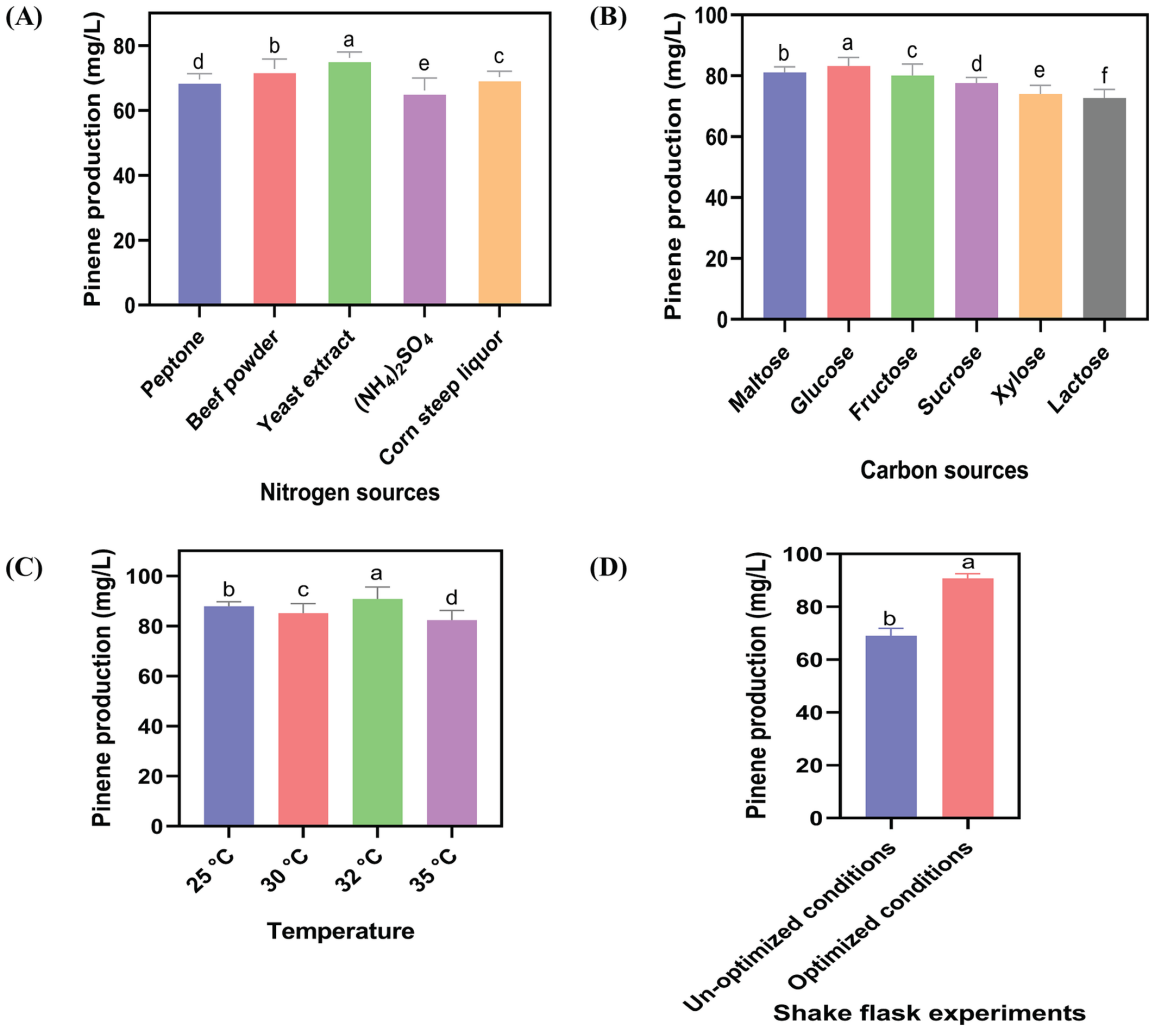
Impact of key fermentation parameters on pinene production in shake flask. **(A)** Impact of five different nitrogen sources on pinene production. **(B)** Impact of six different carbon sources on pinene production. **(C)** The culture was induced with 0.2% arabinose at four different temperatures (25°C, 30°C, 32°C, 35°C). **(D)** Pinene production in unoptimized and optimized conditions by HSY012. OD_600_ was about ∼0.6. All the experiments were performed in triplicates. Data are expressed as the mean values ± standard deviation (*n* = 3). Values in columns with different letters are significantly different at a 5% level, according to Tukey’s Highest Difference Test.

The final strain, HSY012, was further utilized in shake fermentation experiments to produce pinene. The agitation speed, aeration rate, and pH were set at 1.5 vvm, 220 rpm, and 7.2, respectively. Optimization of culture medium and processing conditions can be utilized to increase the quantity and quality of biofuel. Fermentation was carried out using flask equipment set under optimized conditions. These conditions included the cultivation temperature, which needed to strike a balance between cell growth and product formation; the nitrogen source, which could impact the vitality and viability of bacteria; and the carbon source, which influenced the concentration of intracellular acetyl-CoA (Gao et al., 2023; Rajpurohit and Eiteman, 2024; Ayivi et al., 2022). To improve the pinene titers, the “one-factor at-a-time” method was exploited to optimize all the parameters (Figure 6). The highest pinene production (90.76 mg/L) was obtained when the HSY012 strain was grown on a fermentation medium containing 3% glucose and 2.5% yeast extract, with induction using 0.2% arabinose at a temperature of 32°C. Using glucose as a carbon source resulted in the highest pinene yield compared to maltose, fructose, sucrose, xylose, and lactose by HSY012, with maltose being the second-best carbon source.

### 2.4 Optimal conditions for pinene production in a 5-L fermenter

**Figure 7:**
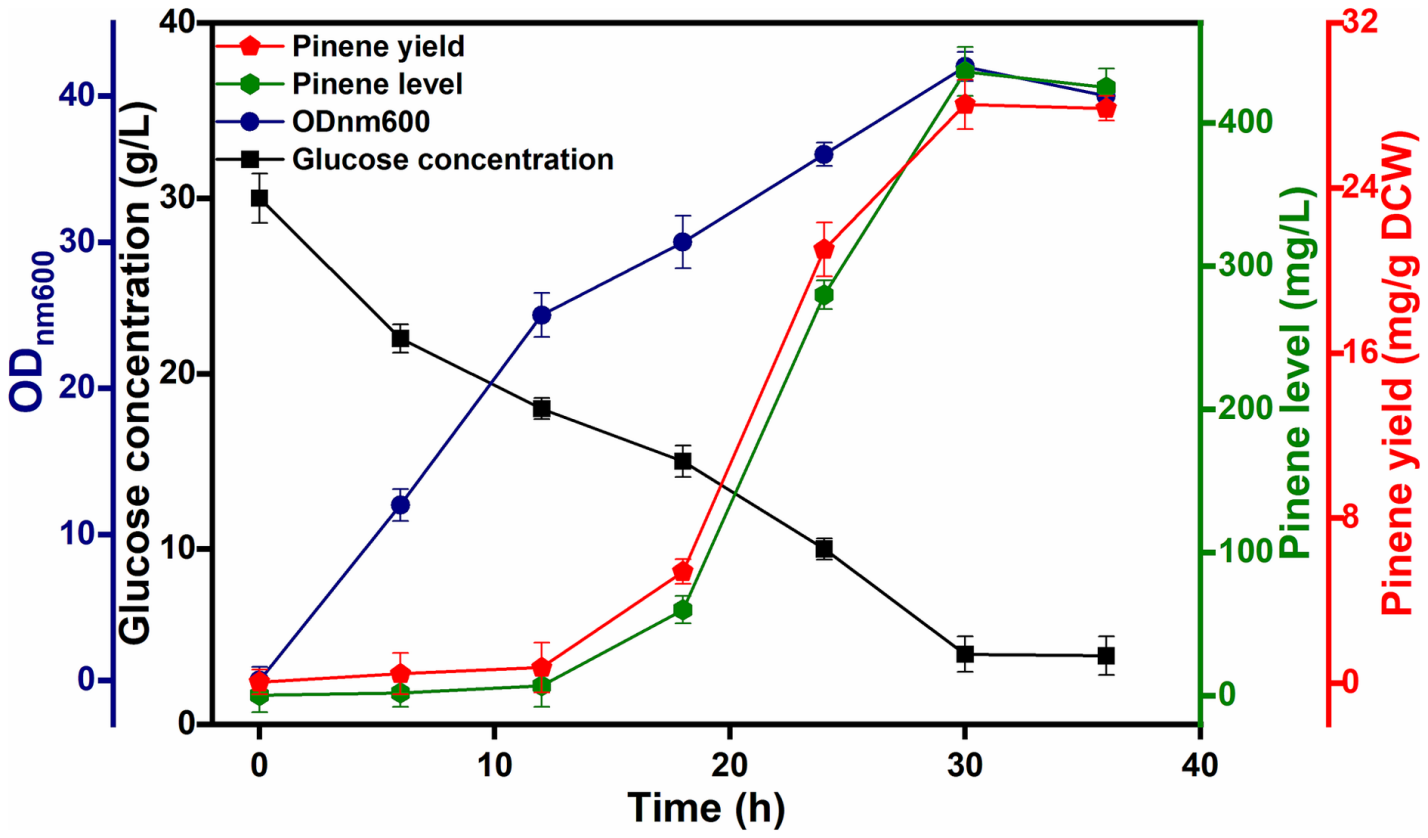
Fermentation in 5 L fermentor depicting pinene level, yield, cell growth, and glucose consumption by HSY012. Hexagon, pentagon, circle, and square” that represent the pinene level, pinene yield, growth ability and glucose concentration. Induction was executed at 32°C, and induction arabinose concentration was 0.2%.

The final strain, HSY012, consisting of a biosynthetic pathway of pinene, accumulated pinene up to 90.76 mg/L under flask fermentation conditions. Afterwards, a 5 L bio-fermentor was used for pinene production by strain HSY012 using the optimized treatment that gave the best results in shake flask experiments. In batch fermentation, glucose as a carbon source, along with yeast extract, was used successfully to obtain better results in terms of pinene production. The induction temperature was fixed at 32°C. Following induction in the bio-fermentor, there was a rapid increase in *α*-pinene production, peaking at approximately 280.78 mg/L between 18 and 24 h. The highest pinene concentration of 436.68 mg/L with a mean productivity of 14.55 mg/L/h was achieved after 30 h culture in a 5-L fermenter. The cell density of the engineered strain reached about 40, while the glucose concentration reduced to 5 g/L after 36 h of fermentation (Figure 7). Besides the unknown mechanism of chromosomal sites (8, 23, 44, 58, and *LpxM*) imparting toxicity stress tolerance to strains, optimizing cofactor metabolic pathways achieves further improvement in pinene production and cell growth. Furthermore, comprehensive analysis based on proteomics, transcriptomics, or metabolomics can help identify major restriction factors such as product synthesis, growth, and cofactor consumption, which can then be targeted for further optimization.

## 3 Discussion

Recently, the development of human society has emerged as a critical global priority, driven by growing concerns over the depletion of fossil fuels and the associated environmental risks (Hussain et al., 2024). Consequently, there has been a significant increase in interest in microbial biofuel production, which is essential for creating sustainable economies and maintaining clean environments. Even though the plasmid expression system is commonly used for microbial pinene production, the pinene level is low. This lower production is thought to result from plasmid instability or the overexpression of mevalonate pathway genes that cause metabolic burden (Troost et al., 2019; Wu et al., 2021). In light of these challenges, we employed CRISPR/Cas9 and lambda Red technology to establish a rational design model that optimizes the copy number of heterogenous expression pathways to achieve sustainable pinene production. Notably, the resulting *E. coli* strain exhibited better stability of pinene productivity compared to strains that relied on plasmids.

This study demonstrated the rational design model’s significance in achieving sustainable pinene production in *E. coli* by optimizing copy number and integration site for a heterogenous pinene expression pathway. Through this approach, the lycopene-producing strain DH411 was genetically modified and produced pinene up to 69.08 mg/L. Subsequently, further enhancement in pinene production (90.76 mg/L) was accomplished through the optimization of the production medium (glucose with yeast extract) and induction temperature (32°C) in the shake flask experiment. Finally, the integrated *Escherichia coli* (HSY012) strain produces 436.68 mg/L pinene in a 5 L fermenter, which was 42.81- and 286.3-fold higher than the previously reported pinene level in the *Saccharomyces cerevisiae* and *Synechococcus* sp. PCC 7002 (Ren et al., 2022; Yang et al., 2021). In comparison to other *E. coli*-produced pinene systems, HSY012’s pinene level was 2.62-fold higher than Niu et al. (2018) integrated strain, and it was 80.5% of Zhou et al. (2023) findings (541.8 mg/L) in batch culture. However, its mean pinene productivity of 14.55 mg/L/h was higher than Zhou et al.’s and Niu et al.’s experimental results by 1.93-fold and 2.44-fold, respectively (Niu et al., 2018; Zhou et al., 2023). Compared to Niu et al. (2018) and Zhou et al. (2023), our strain’s better productivity may be due to the better optimization of the copy number of heterogenous expression pathways and medium composition. Although many studies found the role of integration sites in achieving improved microbial productivity, underlying biological mechanisms leading to the desired phenotype will be further explored (Goodall Emily et al., 2018; Dovala et al., 2016). Meanwhile, our lower pinene production than Zhou et al. (2023) may be due to the difficulty in tuning the expression level of heterologous genes, especially pinene synthase, after chromosomal integration. In our opinion, modifying the promoter and using a fed-batch system can further improve pinene production. Although the pinene production of the HSY012 strain is lower than that of the integrated strain reported by Zhou et al. (2023), it requires less time to reach optimal pinene production during the fermentation process. Our findings highlight that appropriate strain engineering, combined with the optimization of fermentation conditions, can significantly enhance both the yield and productivity of the strain. For future applications in industrial pinene production, we aim to further improve the production and productivity of the HSY012 strain by implementing the aforementioned approaches.

Several physiological parameters, like temperature, nitrogen, and carbon sources, were optimized to increase the pinene titer in *E. coli*. In this regard, it has been observed that in media, different nitrogen sources like yeast extract, peptone, beef powder, corn steep liquor, and (NH_4_)_2_SO_4_ promote cell growth and trigger the synthesis of bioproducts (Rojo et al., 2023). The improved pinene yield when using yeast extract can be attributed to its high vitamin content compared to other nitrogen sources (Elfeky et al., 2019). The overall increase in production is thought to result from a change in the nutrient composition, creating nutritional stress that significantly enhances pinene production by HSY012 (Widadri et al., 2019; Singh et al., 2016). Then, the third parameter, induction temperature, had a significant impact on the pinene production. It is found that the performance of recombinant enzymes can be increased at low induction temperatures mainly due to a decrease in inclusion bodies in recombinant *E. coli* (Mital et al., 2021). Finally, this optimized fermentation parameter increased the final concentration of total pinene.

In this study, the engineered strain demonstrated higher pinene productivity and optimum yield, underscoring its vast potential for being used as a tested strain for industrial trials. Our strain performs well in batch fermentation systems, and utilizing feed-batch fermentation could further increase pinene production. Furthermore, food processing wastes can serve as a feedstock material for sustainable pinene production, further reducing the cost of fermentation. Subsequently, using inexpensive and readily available raw materials potentially impacts the economic feasibility of pinene biosynthesis.

Nonetheless, a significant limitation associated with the production of pinene by *E. coli* pertains to the toxicity caused by pinene and its precursor, geranyl pyrophosphate (GPP). Various approaches should be adopted to counter this problem of pinene toxicity. Several studies have highlighted the significance of different chromosomal regions in determining the physiological capabilities of bacteria, including their metabolic and survival properties (Goodall Emily et al., 2018). Sometimes, the target deletion of non-essential chromosomal regions increases CO_2_ fixation capability and biofuel production in microorganisms (Wang et al., 2021). In the present study, the non-essential regions 8th, 44th, 58th, 23rd, and *LpxM* are used to integrate heterogeneous expression pathways, which is in line with the findings of Dovala et al. (2016). The latter noted that these regions have specialized functions such as enhancing cell growth, preserving bacterial pathogenicity, synthesizing natural metabolites, and achieving tolerance phenotype. Additionally, these regions may be associated with some unknown functions (Dovala et al., 2016). Furthermore, studies have demonstrated that the deletion of *LpxM* sites causes irregularity in macrophage invasion and defective growth in the air sac of chickens (Xu et al., 2013). The presence of *LpxM* sites in *Salmonella typhimurium*, *Acinetobacter baumannii*, and *Escherichia coli* has been found to positively contribute to enhanced outer membrane integrity and induce resistance against cationic antimicrobial peptides (CAMP) (Boll Joseph et al., 2015; Olaitan et al., 2014). These non-essential regions play a pivotal role in enhancing growth rate, biofuel production, and tolerance toward inhibitory end products. These findings hold significance for advancing engineered *E. coli* strains capable of biofuel production. Overall, this conclusion governs further optimization of genetic engineering and omic analysis.

## 4 Methodology

### 4.1 Primers, plasmids, and bacterial strains

The details about plasmids and strains are provided in Table 2. The construction of plasmids was accomplished through *E. coli* DMT, DH5α, and DH1. For eliminating the methylated plasmid *in vivo*, *E. coli* DMT was used. All primers’ manufacturing was performed by the local renowned GENEWIZ company (listed in Supplementary Table S1). Restriction enzymes, PrimeStar Max DNA polymerase, Takara DNA ligation, Clonexpress Ultra cloning, Plasmid miniprep, and Gel extraction kit were used. All operations were performed as per instructions.

**Table 2:** Plasmids and bacterial strains.

Strains	Detail	Reference
DH411	DH06 Δ57th::T7 ES araB::T7RNAP-tetA; Δ64th::T7 S1; Δ23th::T7lac GC	Wei et al. (2018)
DH416	DH414 Δ58th::T7 S1	Hussain et al. (2021)
HSY001	DH416/pETL- PG1	In present study
HSY002	DH416/pETL- PG2	In present study
HSY003	DH416/pETL- PG3	In present study
HSY004	DH411/pETL- PG1	In present study
HSY005	DH411/pETL- PG2	In present study
HSY006	DH411/pETL- PG3	In present study
HSY007	DH411 Δ8th::T7 PG1 (counter-clockwise)	In present study
HSY008	HSY007Δ23th GC	In present study
HSY009	HSY008 Δ*lpxM*::T7 S1	In present study
HY09E	HSY009/pCC1E	In present study
HY09S	HSY009/pCC1S	In present study
HSY010	HSY009 Δ44th::T7 PG1	In present study
HSY011	HSY009 Δ58th::T7 PG1	In present study
HSY012	HSY009 Δ23rd::T7 PG1	In present study
HSY013	HSY012/pCC1P	In present study

Plasmids	Detail	Reference
pET3b	pMB1 origin, T7 promoter; AmpR	Novagen
pETL	pET3b derived, T7lac promoter; AmpR	Our lab
pUC57-G9	pUC57 derived, carrying GPPS gene from *A. grandis*; AmpR	GENEWIZ, Inc.
pUC57-P1	pUC57 derived, carrying PS gene from *A. grandis*; AmpR	GENEWIZ, Inc
pUC57-P2	pUC57 derived, carrying (+) PS gene from *P. taeda*; AmpR	GENEWIZ, Inc
pUC57-P3	pUC57 derived, carrying (−) PS gene from *P. taeda*; AmpR	GENEWIZ, Inc
pUC57-PG1	pUC57 derived, carrying PG1; AmpR	GENEWIZ, Inc
pUC57-PG2	pUC57 derived, carrying PG2; AmpR	GENEWIZ, Inc
pUC57-PG3	pUC57 derived, carrying PG3; AmpR	GENEWIZ, Inc
pETL-PG1	pETL derived, carrying PG1; AmpR	In present study
pETL-PG2	pETL derived, carrying PG2; AmpR	In present study
pETL-PG3	pETL derived, carrying PG3; AmpR	In present study
pET3b-PG1	pET3b derived, carrying PG1; AmpR	In present study
pCC1E	pCC1FOS derived, carrying T7 ES; CmR	(Hussain et al., 2021)
pCC1S	pCC1FOS derived, carrying T7 S1; CmR	(Wei et al., 2018)
pCC1P	pCC1FOS derived, carrying T7 PG; CmR	In present study
pETE	pET3b derived, carrying T7 ES; AmpR	Our lab
pETS	pET3b derived, carrying T7 S1; AmpR	(Wei et al., 2018)
pETP	pET3b derived, carrying T7 PG; AmpR	In present study
pCNA	pKOBEG derived, carrying I-CreI endonuclease gene; AmpR	Our laboratory
pSNA	pKOBEG derived, carrying I-SceI endonuclease gene; AmpR	Our laboratory
pSNK	pKOBEG derived, carrying I-SceI endonuclease gene; KanR	Our laboratory
pBDC	p15A origin, sacB cassette; CmR	Our laboratory
pBDK	p15A origin, sacB cassette; KanR	Our laboratory
pBDC-58i	pBDC derived, carrying 58th homologous region; CmR	Our laboratory
pKIS	pMB1 origin, T7 S1, I-SceI; KanR	(Wei et al., 2018)
pCas9	p15A origin, Ptet Cas9; CmR;TetR	(Jiang et al., 2015)
pRNA	pSC101 ori, Ptet sgRNA, araC-PBAD gam-bet-exo; TetR	(Reisch and Prather, 2015)
pKD-15A	pRNA derived, sgRNA-p15A ori	(Reisch and Prather, 2015)
pRNA-8r	pRNA derived, sgRNA-eighth homologous region	Our laboratory
pRNA-GC	pRNA derived, sgRNA-GC	Our laboratory
pCP-8r	p15A origin, T7 PG1, carrying eighth homologous region; CmR	Our laboratory
pCP-23d	p15A origin, carrying 23th homologous region; CmR	Our laboratory

PG1 (*A. grandis* GPPS-*A. grandis* PS); PG2 (*A. grandis* GPPS- *P. taeda* (+) PS); PG3 (*A. grandis* GPPS- *P. taeda* (−) PS); PG (GPPS-PS); GC (*A. fulgidus* idsA- *P. agglomerans* crtI-crtB); ES (*E. faecalis mvaE-mvaS*); S1 (*S. pneumoniae* mvk-mvaK2-mvd1-fni).

### 4.2 Construction method of the engineered strain

The engineered strains DH411, DH416, and HSY009 were constructed via the lambda-Red recombination method. Moreover, the remaining other strains were constructed via CRISPR/Cas9/lambda-Red techniques. Using *E. coli* DH411 as a host, the expression element PG1 was integrated counterclockwise into the 8th region to generate the strain HSY007/pCas9. Using plasmid pCP-8r as a template, PCA3rc and PCA5arc as primers, the target fragment containing homologous region 8 and expression element PG1 was amplified by PCR at about 4.1 kb (see Supplementary Table S2 for CRISPR plasmids). Recombination utilizes plasmids pCas9 and pRNA-8r. After that, *E. coli* HSY007 as a host, the 23 region was knocked out to construct the strain HSY008. Using plasmid pCP-23d as template, PCA3rc, and PCA5rc as primers, a target fragment containing the homologous region of region 23 was amplified by PCR at about 1.1 kb. Following that, recombination was carried out utilizing plasmids pCas9 and pRNA-23d. To eliminate the plasmid pCas9 in the host, plasmid pKD-15A was introduced into the host. Dehydrytetracycline was added to induce the expression and cleavage of pCas 9 and sgRNA, respectively. Furthermore, *E. coli* HSY008 was used as the host, and expression element S1 was integrated into the *LpxM* locus clockwise to generate the strain HSY009. In the next step, plasmids pBDC-58i and pCNA were recombined, followed by the second step of recombination using plasmids pSNA and pKILS. The first step of recombination employed primers ES-0 and ES-1; while the second step of recombination was verified by PCR using primers M-0 and M-1. Lastly, *E. coli* HSY009 was used as the host, and the expression element PG1 was integrated sequentially into the 44th, 58th, and 23rd locus to generate three strains HSY010, HSY011, and HSY012, respectively.

### 4.3 Construction of plasmids

Three heterogeneous expression cassettes were utilized: ES cassette, S1 cassette, and PG cassette. Each cassette consists of specific genes obtained from different organisms. The ES cassette consists of two genes *mvaE* and *mvaS* isolated from *Enterococcus faecalis*. This cassette is responsible for catalyzing the conversion of acetyl-CoA into mevalonic acid. It is commonly referred to as the upper MVA pathway. The S1 cassette consists of genes *mvaK2*, *mvd1*, *fni*, and *mvk* isolated from *Streptococcus pneumoniae*. This cassette catalyzes the reaction to convert mevalonic acid to IPP and DMAPP. These compounds serve as the major precursors for the enhanced production of terpenoids. The S1 cassette is commonly named the lower MVA pathway. The third expression cassette, PG cassette, consists of two genes, *GPPS* and *PS*, which were isolated from *Abies grandis*. The PG cassette is responsible for the production of pinene, which is the target product of interest (see Supplementary Table S3).

In this study, two plasmids, pRNA-8r and pRNA-GC, were generated from pRNA (Reisch and Prather, 2015). The fragment F1 (3.5 kb) was amplified by PCR using primers 8F and BetaR and pRNA as a template. Similarly, the fragment F2 (3.5 kb), was amplified by PCR using primers pkdseq1 and 8R. These two PCR products, F1 and F2, were subsequently assembled to create the plasmid pRNA-8r. RNA8-1 and BetaR. PCR primers RNA8-1 and BetaR were used to confirm the presence of positive pRNA-8r colonies. The first fragment was amplified by PCR using primers 23 GF and BetaR and pRNA as a template, while the second fragment was amplified by PCR using primers pkdseq1 and 23G. These two PCR products were assembled to create the plasmid pRNA-GC. PCR identification was conducted using primers RNA-23G and BetaR.

Additionally, the donor plasmids pCP-8r and pCP-23d were employed in this study. The 2.2 kb backbone CP containing cat and p15A ori was amplified by PCR using primers PCA5 and PCA3 and pACYCDuet-1 as a template. The PG fragment containing element PG1 was amplified by PCR using primers TE5 and TE3 and pET3b-PG1 as templates. The *E. coli* DH1 genome was used as a template, the 0.6 kb 8 L fragment containing the left homology region was amplified by PCR using primers L8-5 and L8-3; while the 0.6 kb 8R fragment containing the right homology region was amplified by PCR using primers R8-5 and R8-3, respectively. Four PCR products, CP, PG, 8 L, and 8R were assembled to create the plasmid pCP-8r.

The left homologous region of the 23 region was amplified by PCR (about 500 bp) using primers L23-5 and L23d-3 and the *E. coli* DH1 genome as a template. The right homologous region (about 500 bp) of the 23 region was amplified by PCR using primers R23d-5 and R23-3 and *E. coli* DH1 genome as a template. Plasmid pCP-23d was constructed after recombination of cytoskeleton CP, left homologous fragment of 23 region, and right homologous segment of 23 region. The target fragments for homologous recombination were amplified by PCR using primers PCA3rc and PCA5rc and plasmids pCP-8r or pCP-23d as a template.

Two primers RP5 and FP3 were used to amplify the backbone of pCC1P (fragment CC1) and plasmid pCC1FOS was used as the template. The fragment PGCC, consisting of T7 PG flanked by 15–20 bp homologous region of fragment CC1 was amplified from plasmid pETP with the assistance of two primers BI5 and BI3, respectively. Afterwards, the cloning kit (TaKaRa Co.) was used to assemble two fragments, named CC1 and PGCC to generate plasmid pCC1P which was identified by PCR via two primers, namely FP-0 and RP-1, respectively.

The isolated GPPS gene fragment was digested from pUC57-GS with Xba I and BamH I, followed by insertion into Spe I/BamH I sites of plasmid pUC57-PS to create pUC57-GS-PS. The isolated PG fragment was digested from pUC57-GS-PS with Xba I and BamH I and then ligated into the corresponding sites of pET3b to create pETP.

The PG1, PG2, and PG3 fragments were digested from pUC57-PG1, pUC57-PG2, and pUC57-PG3 and inserted one by one into plasmid pETL to create pETL-PG1, pETL-PG2, pETL-PG3. The PG1 fragment was inserted in pET3b to create pET3b-PG1. The primers used to amplify the PG1 fragment from pUC57-PG1 were RT5 and RT3, primer FT5 and FT3 for the PG2 fragment, and primer HT5 and HT3 for the PG3 fragment, respectively.

### 4.4 Inoculation preparation and fermentation for pinene production

For culture, the single colony of strain HSY012 was grown overnight at 32°C and 220 rpm in 100 mL of LB medium containing NaCl (10 g/L), tryptone (10 g/L), and yeast extract (5 g/L). Then, the inoculation was carried out in 50 mL of the medium at an optical density (OD_600_) of 0.1. The fermentation medium consisted of 5 g/L of yeast extract, 5.0 g/L of NaH_2_PO_4_.2H_2_O, 7 g/L of K_2_HPO_4_.3H_2_O, 2.5 g/L of NaCl, 5 g/L of Tween 80, 10 g/L of glycerol, 0.5 g/L of MgSO_4_ and 10 g/L of glucose (Hussain et al., 2021). The culture system was induced with 0.2% of L-arabinose when the OD_600_ reached ∼0.6–0.8. For strain HSY012, fermentation was carried out in a 5 L fermenter with an initial working volume of 3 L. The system was induced with 0.2% of L-arabinose after 7 h. During fermentation, the optimum temperature and pH for culture incubation were 32°C and 7 (controlled by NaOH). Optimal pinene production was obtained at 220 rpm agitation and 1.5 vvm aeration rate. The fermentation medium was supplemented again with L-arabinose (0.2%) after 12 h (Xiong et al., 2015). Furthermore, a spectrophotometer evaluated the bacterial growth by measuring the OD_600_. At the start of the experiment, 15% (6 mL) of dodecane, purchased from Skyrun Co., Ltd, was poured into a fermentation medium through a 5 mL pipette. It was applied as an overlay to trap the pinene. This chemical is kept at room temperature.

### 4.5 Optimization of pinene production at flask scale

The effects of three factors, such as carbon source, nitrogen source, and temperature, on pinene production were studied. The fermentation medium was supplemented individually with five different nitrogen sources (peptone, beef powder, yeast extract, ammonium sulfate, and corn steep liquor) at a concentration of 2.5%, followed by the addition of different carbon sources (maltose, glucose, fructose, sucrose, xylose, and lactose), with the concentration of 3%. The effect of different temperatures (25°C, 30°C, 32°C, 35°C) on pinene production was also studied.

### 4.6 Analysis for pinene determination

For the quantification of pinene, 1.5 mL of microcentrifuge tube carrying 500 *μ*L of the dodecane layer was centrifuged (25,000 *g*, 1 min). Afterwards, the dilution of 50 *μ*L of dodecane was carried out in 450 *μ*L of ethyl acetate that spiked with the internal standard cyclohexanone. GC/MS used a standard curve of *α*-pinene (Sigma Aldrich) to analyze the samples. Agilent 7,890 GC system with 5,975 MSD was used. 19091S-433 Agilent chromatographic column thickness was 0.25 mm, and the length was 30 m. The inlet temperature was 300°C, flow was at 1 mL/min, shunt ratio was at 100: 1, and injection amount was 0.2 L. The oven temperature was initially set at 50°C for 30 s. Subsequently, there was a ramping phase with a rate of 40°C/min, increasing the oven temperature from 50°C to 70°C. Following this, there was another ramping phase at a rate of 25°C/min, raising the temperature from 70°C to 250°C. The final temperature of 250°C was held for 5 min. The reference retention time for cyclohexanone and pinene was 3.8 min and 4.7 min, respectively. All experiments were done with triplicates, and the error bar showed the standard deviation. The standard curve was plotted with the concentration of reference substance as the abscissa, and the ratio of peak heights of pinene to cyclohexanone was plotted on the ordinate (y-axis), as shown in Supplementary Figure S2.

## Data Availability

The original contributions presented in the study are included in the article/Supplementary material, further inquiries can be directed to the corresponding author/s.
