# Supplementary material for: Rational engineering of Escherichia coli strain for stable and enhanced biosynthesis of pinene
Source: Front Microbiol. 2025 Jan 7;15:1527113. doi: 10.3389/fmicb.2024.1527113 (PMC11747701; doi:10.3389/fmicb.2024.1527113)
Supplement: Supplementary file 1 [file Table_1.DOCX]

Supplementary information


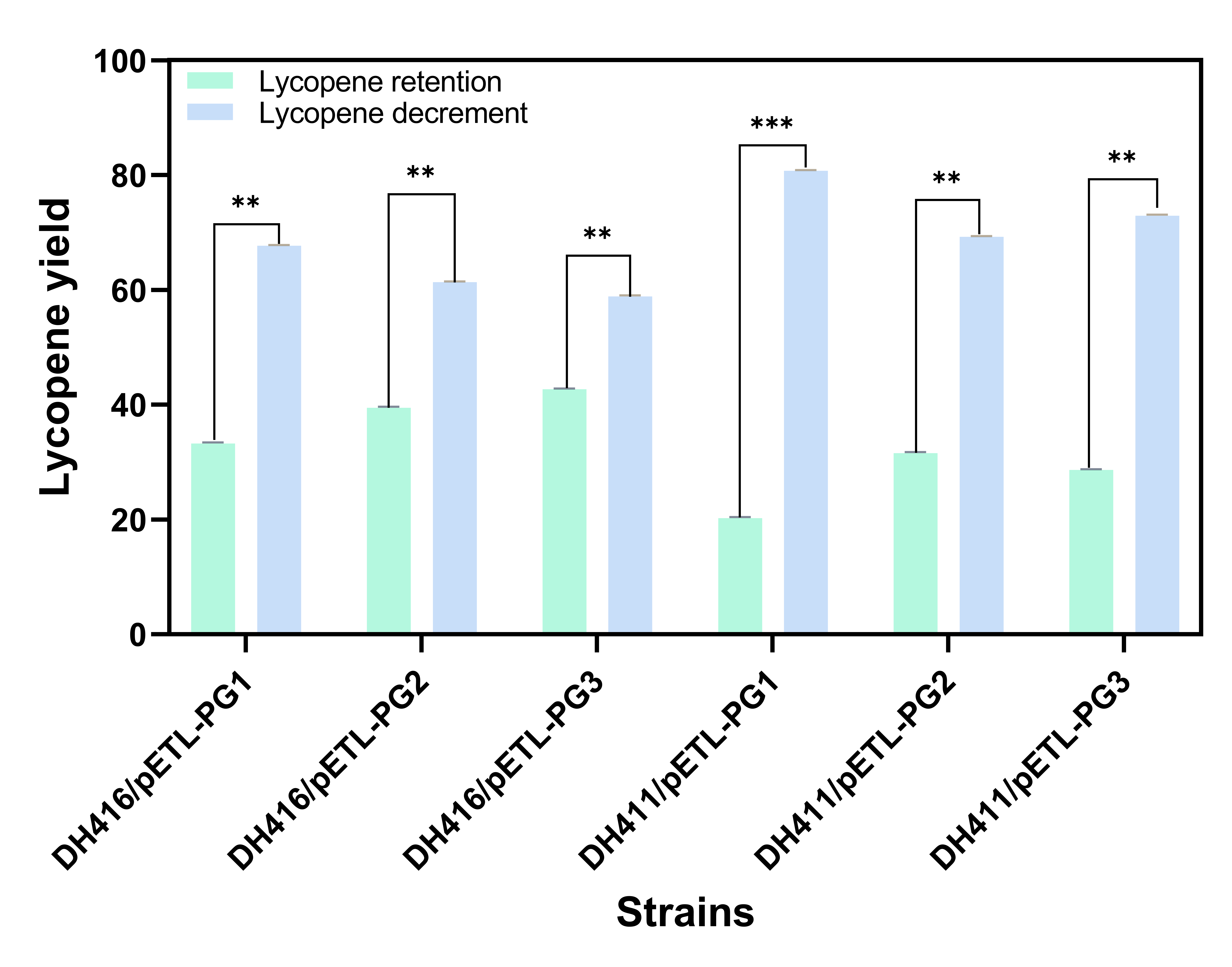


Figure S1. It depicts the lycopene yield of E. coli after the transformation of the pinene expression pathway. Also, it shows that lycopene contents (*p < 0.05, **p < 0.01 ***p > 0.001 indicate statistical significance levels from a t test).

To determine the optimal pathway for pinene synthesis, three plasmids (pETL-PG1, pETL-PG2, or pETL-PG3) were transformed into lycopene-producing strain DH416 and three in strain DH411. The pinene synthesis pathway competes with lycopene synthesis pathways to consume substrates IPP and DMAPP. Strain DH416 and DH411 transformed with plasmids carrying pinene synthesis pathway resulted in reduced lycopene yield. The more lycopene yield decreases, the corresponding pinene synthetic pathway is considered to be more appropriate.


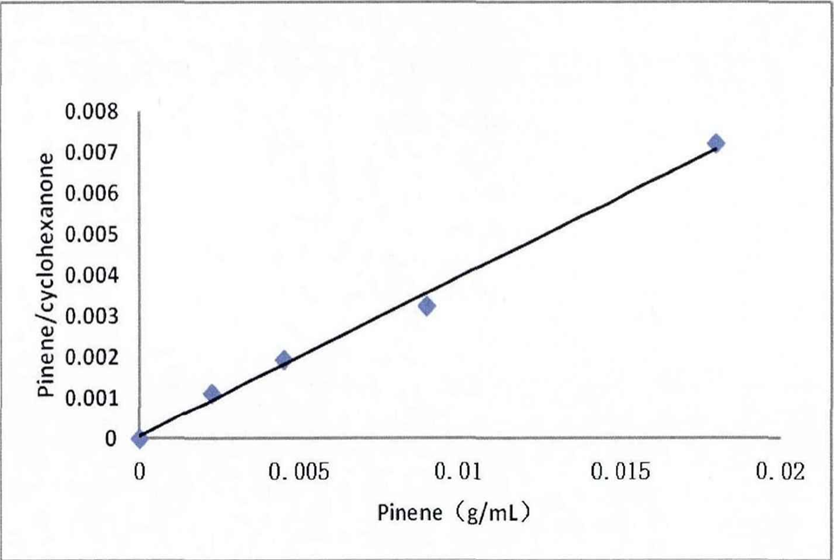


Figure S2. Standard curve of pinene.

The standard curve equation is: y = 0.03899x + 6e^-5^, R^2^ = 0.9942.

Table S1. Primers used in this study

| Primer name | Sequence | Reference |
| --- | --- | --- |
| PCA3rc | AAGGGCATCGGTCGGAT | This study |
| PCA5arc | ACTACGGGAAGGTGA | This study |
| PCA5rc | ACTACCGGAAGCAGTGTGA | This study |
| ES-0 | ACGCTGAACTGCT | This study |
| ES-1 | GAAGTGGTGCCAAC | This study |
| M-0 | AGACATTAAGACTGCGGT | This study |
| M-1 | ATATCACCCAACTGGC | This study |
| 8F | CGACGATAACGCGGCTGAACGTTTTAGAGCTAGAAATAGCAAG | This study |
| BetaR | TTTATAACCTCCTTAGAGCTCGA | This study |
| pkdseq1 | CCAATTGTCCATATTGCATCA | This study |
| 8R | GTTCAGCCGCGTTATCGTCGGTGCTCAGTATCTCTATCACTGA | This study |
| RNA8-1 | TAATGGCGGTGCTGTGACTC | This study |
| 23 GF | CAAAGCCAAGGAAACACTGCGTTTTAGAGCTAGAAATAGCAAG | This study |
| 23GR | GCAGTGTTTCCTTGGCTTTGGTGCTCAGTATCTCTATCACTGA | This study |
| RNA-23G | CAAAGCCAAGGAAACACTGC | This study |
| PCA5 | TCACACTGCTTCCGGTAGT | This study |
| PCA3 | ATCTCGACCGATGCCCTT | This study |
| TE5 | AAGGAGATGGCGCCCAAC | This study |
| TE3 | CCGGATATAGTTCCTCCTTT | This study |
| L8-5 | GGCATCGGTCGAGATAGGCTACCGTCCGCAGTT | This study |
| L8-3 | GGGCGCCATCTCCTTTAATC CCTTGTGACGTAAAAAC | This study |
| R8-5 | GAGGAACTATATCCGGCGAAGGCTGTTTACTTTACTAG | This study |
| R8-3 | CCGGAAGCAGTGTGAGACGCATCAGAGCATCAAC | This study |
| L23-5 | GGCATCGGTCGAGATGGATGCTGATTCCCTGTTCT | This study |
| L23d-3 | GAGGTTTTAACGGCGGATAGTCGTACACAGTTCAC | This study |
| R23d-5 | CGCCGTTAAAACCTCCCTA | This study |
| R23-3 | CCGGAAGCAGTGTGAATCGCATCGTGCTGAAGG | This study |
| RP5 | CTTGGCGTAATCATGG GCTTTTCCAGTTTCGGATAAG | (Hussain et al., 2021) |
| FP3 | GTCGTGACTGGGAAAACGAAATCCAGCCGTATAAGCG | (Hussain et al., 2021) |
| BI5 | GTTTTCCCAGTCACGACAAGGAGATGGCGCCCAA | (Hussain et al., 2021) |
| BI3 | CCATGATTACGCCAAGCCGGATATAGTTCCTCCTTT | (Hussain et al., 2021) |
| FP-0 | GATGTGCTGCAAGGCGATT | (Hussain et al., 2021) |
| RP-1 | CTCGTATGTTGTGTGGAATTG | (Hussain et al., 2021) |
| RT5 | GCCCATGGTATGGAGGAATG | This study |
| RT3 | CAGCTTTCATGCTGCCGCTG | This study |
| FT5 | GCGAGCTCGCGCTAGCAG | This study |
| FT3 | CGACGTCGACGCGGATCC | This study |
| HT5 | CGCGGATCCATGCGCATTG | This study |
| HT3 | GCTGCCGCTGCCGCTGCCGT | This study |

Table S2. CRISPR plasmids used in this study

| **Plasmid used for integration** | |  |  |
| --- | --- | --- | --- |
| **Helper plasmid** | **Sequence** | | **Reference** |
| pCNA | pKOBEG derived, carrying I-CreI endonuclease gene at Nhe I site; AmpR | | Our laboratory |
| pSNA | pKOBEG derived, carrying I-SceI endonuclease gene at Nhe I site; AmpR | | Our laboratory |
| pSNK | pKOBEG derived, carrying I-SceI endonuclease gene at Nhe I site; KanR | | Our laboratory |
| **Landing pad plasmid** |  | |  |
| pBDC | p15A origin, sacB cassette, I-CreI sites; CmR | | Our laboratory |
| pBDK | p15A origin, sacB cassette, I-CreI sites; KanR | | Our laboratory |
| pBDC-8ri | pBDC derived, carrying eighth homologous region; CmR | | Our laboratory |
| pRNA-8 | pRNA-X derived, carrying targeting 8th sgRNA; SpecR | | This study |
| **Donor plasmid** |  | |  |
| pETI | pET3b derived, I-SceI sites; AmpR | | This study |
| pETLI | pET3L derived, I-SceI sites; AmpR | | This study |
| pCP-8r | p15A origin, T7 PG1, 8th homologous region; CmR | | This study |
| pCP-23d | p15A origin, 23th homologous region; CmR | | This study |

Table S3. Sequence of genes.

| Gene | DNA sequence |
| --- | --- |
| fni | TCTAGAAATAATTTTGTTTAACTTTAAGAAGGAGATATACCATGACGACCAACCGCAAAGACGAACACATCCTGTATGCCCTGGAACAGAAAAGCTCCTATAACTCATTTGACGAAGTGGAACTGATTCATAGCTCTCTGCCGCTGTATAACCTGGATGAAATCGACCTGAGTACCGAATTTGCAGGCCGCAAATGGGATTTTCCGTTCTACATTAATGCTATGACGGGCGGTTCCAACAAAGGTCGTGAAATCAATCAGAAACTGGCGCAAGTGGCCGAAACCTGCGGCATTCTGTTTGTTACGGGTTCATATTCGGCGGCCCTGAAAAACCCGACCGATGACAGCTTCTCTGTCAAAAGTTCCCACCCGAACCTGCTGCTGGGCACCAATATTGGTCTGGATAAACCGGTTGAACTGGGCCTGCAGACGGTCGAAGAAATGAACCCGGTGCTGCTGCAGGTGCATGTTAATGTCATGCAAGAACTGCTGATGCCGGAAGGTGAACGTAAATTTCGCAGTTGGCAGTCCCACCTGGCCGATTATAGTAAACAAATTCCGGTGCCGATCGTTCTGAAAGAAGTGGGCTTTGGTATGGATGCAAAAACCATCGAACGTGCGTATGAATTTGGCGTCCGTACCGTGGACCTGTCAGGTCGTGGCGGTACGTCGTTCGCGTATATTGAAAACCGTCGCAGCGGCCAGCGCGATTACCTGAATCAGTGGGGTCAATCTACCATGCAGGCGCTGCTGAACGCCCAAGAATGGAAAGATAAAGTGGAACTGCTGGTTTCTGGCGGTGTCCGCAATCCGCTGGACATGATTAAATGTCTGGTTTTCGGCGCAAAAGCTGTCGGTCTGAGCCGTACCGTGCTGGAACTGGTTGAAACCTACACGGTTGAAGAAGTCATTGGCATCGTGCAGGGTTGGAAAGCGGATCTGCGTCTGATTATGTGCAGCCTGAACTGTGCAACGATCGCTGATCTGCAGAAAGTTGACTATCTGCTGTACGGCAAACTGAAAGAAGCGAATGACCAAATGAAAAAAGCCTAAACTAGTCGCGCGGGATCC |
| mvk | TCTAGAAATAATTTTGTTTAACTTTAAGAAGGAGATATACCATGACTAAAAAAGTGGGTGTTGGGCAAGCACATTCAAAAATTATTCTGATTGGCGAACATGCGGTTGTCTATGGCTATCCGGCGATTAGTCTGCCGCTGCTGGAAGTTGAAGTCACCTGCAAAGTGGTTCCGGCGGAATCCCCGTGGCGTCTGTATGAAGAAGATACCCTGTCAATGGCGGTCTATGCCTCGCTGGAATACCTGAACATCACGGAAGCGTGCATTCGTTGTGAAATCGACAGCGCTATTCCGGAAAAACGCGGTATGGGCAGCTCTGCGGCCATTTCTATCGCAGCTATTCGTGCAGTTTTTGATTATTACCAGGCTGATCTGCCGCATGACGTGCTGGAAATCCTGGTTAACCGCGCAGAAATGATTGCTCACATGAATCCGAGTGGTCTGGATGCCAAAACCTGTCTGTCCGACCAGCCGATTCGTTTTATCAAAAACGTGGGCTTCACGGAACTGGAAATGGATCTGAGTGCATATCTGGTCATCGCTGACACCGGTGTGTACGGCCATACGCGCGAAGCAATTCAGGTCGTGCAAAATAAGGGTAAAGATGCGCTGCCGTTCCTGCACGCCCTGGGTGAACTGACCCAGCAAGCAGAAATTGCTATCTCACAGAAAGACGCGGAAGGTCTGGGCCAGATCCTGTCGCAAGCCCATCTGCACCTGAAAGAAATTGGTGTTAGTTCCCTGGAAGCGGATAGCCTGGTCGAAACGGCACTGTCTCATGGTGCGCTGGGTGCAAAAATGTCTGGCGGTGGCCTGGGTGGCTGCATTATCGCACTGGTGACCAATCTGACGCACGCGCAGGAACTGGCAGAACGCCTGGAAGAAAAAGGCGCGGTTCAAACCTGGATTGAAAGCCTGTAAACTAGTCGCGCGGGATCC |
| mvaK2 | TCTAGAAATAATTTTGTTTAACTTTAAGAAGGAGATATACATATGATTGCTGTGAAAACCTGTGGTAAACTGTACTGGGCGGGCGAATACGCTATCCTGGAACCGGGTCAACTGGCACTGATTAAAGACATTCCGATCTATATGCGTGCAGAAATTGCTTTTAGTGATTCCTATCGCATCTACTCCGATATGTTTGACTTCGCGGTTGATCTGCGTCCGAATCCGGACTATTCACTGATTCAGGAAACCATCGCGCTGATGGGCGATTTTCTGGCCGTGCGTGGTCAAAACCTGCGCCCGTTCTCGCTGGCCATTTACGGCAAAATGGAACGCGAAGGTAAAAAATTTGGTCTGGGCAGCTCTGGCTCAGTGGTTGTCCTGGTGGTTAAAGCGCTGCTGGCCCTGTATAATCTGAGCGTGGATCAGAACCTGCTGTTCAAACTGACCTCTGCGGTTCTGCTGAAACGTGGTGATAATGGCAGTATGGGTGACCTGGCCTGCATTGCGGCCGAAGACCTGGTCCTGTACCAGAGCTTTGACCGTCAAAAAGTGGCAGCTTGGCTGGAAGAAGAAAACCTGGCAACGGTTCTGGAACGCGATTGGGGCTTTTCAATCTCGCAGGTTAAACCGACCCTGGAATGTGACTTCCTGGTCGGTTGGACGAAAGAAGTTGCCGTCAGTTCCCACATGGTCCAGCAAATCAAACAGAACATCAACCAAAACTTCCTGACCTCATCGAAAGAAACGGTCGTGTCACTGGTGGAAGCACTGGAACAGGGCAAATCGGAAAAAATTATCGAACAAGTGGAAGTTGCTAGTAAACTGCTGGAAGGTCTGTCCACCGATATTTATACGCCGCTGCTGCGCCAGCTGAAAGAAGCATCTCAGGACCTGCAAGCGGTGGCCAAAAGCTCTGGCGCTGGCGGTGGCGATTGCGGTATCGCACTGAGCTTCGACGCTCAGTCTACCAAAACGCTGAAAAATCGTTGGGCGGATCTGGGCATTGAACTGCTGTACCAGGAACGCATCGGTCACGATGACAAAAGTTAAACTAGTCGCGCGGGATCC |
| mvd1 | TCTAGAAATAATTTTGTTTAACTTTAAGAAGGAGATATACCATGTACCACAGCCTGGGCAATCAGTTTGACACCCGCACCCGCACCTCACGTAAAATCCGCCGCGAACGCTCTTGTTCAGATATGGACCGTGAACCGGTGACCGTTCGCAGTTATGCGAACATTGCCATTATCAAATACTGGGGCAAGAAAAAAGAAAAAGAAATGGTGCCGGCCACCAGCTCTATCAGTCTGACGCTGGAAAACATGTACACCGAAACCACGCTGTCCCCGCTGCCGGCAAATGTCACCGCAGATGAATTTTACATTAACGGTCAGCTGCAAAATGAAGTGGAACATGCTAAAATGAGCAAAATTATCGATCGTTATCGCCCGGCAGGCGAAGGTTTCGTTCGTATCGACACGCAGAACAATATGCCGACCGCAGCAGGTCTGAGTTCCTCATCGAGCGGTCTGTCAGCGCTGGTCAAAGCGTGCAATGCTTATTTTAAACTGGGCCTGGATCGTTCGCAGCTGGCGCAAGAAGCCAAATTTGCAAGTGGTTCTAGTTCCCGCTCCTTCTATGGTCCGCTGGGTGCATGGGATAAAGACAGCGGCGAAATTTACCCGGTGGAAACGGATCTGAAACTGGCGATGATTATGCTGGTTCTGGAAGATAAGAAAAAACCGATCTCATCGCGTGACGGTATGAAACTGTGTGTTGAAACCAGCACCACGTTTGATGACTGGGTCCGCCAGTCTGAAAAAGATTACCAAGACATGCTGATCTACCTGAAAGAAAACGATTTCGCGAAAATCGGCGAACTGACCGAGAAAAACGCGCTGGCTATGCACGCGACCACGAAAACGGCTAGCCCGGCGTTTTCTTATCTGACCGATGCTTCATACGAAGCGATGGACTTCGTGCGTCAGCTGCGCGAAAAAGGCGAAGCCTGCTATTTTACGATGGATGCAGGTCCGAACGTCAAAGTGTTCTGTCAGGAAAAAGACCTGGAACACCTGTCTGAAATTTTCGGTCAACGTTACCGCCTGATCGTTTCAAAAACCAAAGACCTGTCGCAGGATGACTGCTGTTAAACTAGTCGCGCGGGATCC |
| idsA | ATGGATAGATTTCAGCGTTTGATTGCCATGCTGAAGGAGGAAATTGCGAAACGTGCCGAAATTATCAACAAAGCCATTGAAGAGCTTCTGCCGGAACGTGAGCCGATTGGTCTCTACAAAGCCGCACGTCATCTGATCAAAGCAGGTGGCAAGCGTCTGCGTCCTGTAATCAGCCTCTTAGCAGTCGAAGCCCTTGGTAAAGACTACAGAAAGATTATCCCGGCTGCTGTCAGCATTGAAACAATCCACAACTTCACCCTCGTGCATGACGACATCATGGACCGTGACGAGATGCGTCGTGGTGTTCCGACTGTACACAGAGTTTATGGTGAAGCGACTGCCATTTTAGCAGGCGACACACTCTTTGCTGAAGCCTTCAAGCTGCTGACAAAGTGCGATGTTGAGAGCGAGGGTATCAGAAAAGCTACAGAAATGCTTTCGGACGTTTGCATTAAAATTTGCGAGGGTCAGTACTACGACATGAGCTTTGAGAAAAAGGAGAGCGTTTCCGAGGAGGAGTATCTCAGAATGGTCGAGCTGAAGACCGGTGTGCTGATTGCAGCTTCTGCAGCATTACCTGCGGTGCTTTTTGGTGAGAGCGAGGAAATTGTAAAGGCGCTGTGGGACTACGGTGTTCTTAGCGGTATTGGCTTCCAGATCCAGGACGACCTGCTTGACCTGACTGAGGAGACCGGTAAGGACTGGGGTAGCGACCTGCTTAAAGGTAAGAAAACCCTGATTGTCATTAAGGCGTTCGAAAAGGGTGTGAAGCTGAAGACATTTGGTAAGGAAAAGGCGGACGTCTCTGAGATTAGAGATGATATCGAAAAGTTAAGAGAGTGTGGTGCGATTGATTACGCTGCCAGCATGGCAAGAAAGATGGCTGAAGAGGCGAAAAGAAAGCTCGAAGTTCTGCCTGAAAGCAAAGCCAAGGAAACACTGCTGGAACTTACCGACTTCTTGGTTACAAGAAAAAAGTAA |
| GPPS | ATGCGTTTCGATTTTAACAAATACATGGACTCAAAAGCAATGACCGTGAATGAAGCCCTGAACAAAGCTATCCCGCTGCGCTATCCGCAGAAAATCTATGAATCAATGCGTTACTCGCTGCTGGCCGGCGGTAAACGTGTTCGCCCGGTCCTGTGCATTGCGGCCTGTGAACTGGTGGGCGGCACCGAAGAACTGGCGATCCCGACGGCGTGCGCCATTGAAATGATCCATACCATGTCACTGATGCACGATGACCTGCCGTGTATTGATAACGATGACCTGCGTCGCGGCAAACCGACGAATCATAAAATCTTTGGCGAAGACACCGCAGTCACGGCTGGTAACGCGCTGCATTCCTATGCCTTCGAACACATTGCAGTGAGTACCTCCAAAACGGTTGGCGCGGATCGTATCCTGCGCATGGTTAGCGAACTGGGTCGTGCAACCGGCTCCGAAGGTGTCATGGGTGGTCAGATGGTGGATATTGCAAGCGAAGGTGATCCGTCTATCGACCTGCAAACCCTGGAATGGATTCATATCCACAAAACGGCGATGCTGCTGGAATGCTCAGTGGTTTGTGGCGCTATTATCGGCGGTGCGTCGGAAATTGTTATCGAACGTGCCCGTCGCTATGCACGCTGCGTCGGTCTGCTGTTTCAGGTCGTGGATGACATTCTGGATGTTACCAAAAGCTCTGACGAACTGGGCAAAACGGCCGGTAAAGACCTGATCAGCGACAAAGCAACCTACCCGAAACTGATGGGCCTGGAAAAAGCTAAAGAATTTAGCGATGAACTGCTGAACCGTGCAAAAGGCGAACTGTCTTGTTTCGATCCGGTGAAAGCAGCTCCGCTGCTGGGTCTGGCTGACTACGTTGCGTTCCGCCAAAATTAA |
| PS | ATGGCACGTCGCGGCAAAAGCATTACCCCGAGTATTAGTATGTCCAGCACCACGGTCGTTACGGATGATGGCGTTCGTaGacGtATGGGTGATTTCCACTCCAACCTGTGGGACGACGATGTTATCCAGTCTCTGCCGACCGCCTATGAAGAAAAAAGTTATCTGGAACGTGCAGAAAAACTGATCGGTGAAGTTAAAAACATGTTCAACAGTATGTCCCTGGAAGATGGCGAACTGATGTCGCCGCTGAACGACCTGATCCAGCGCCTGTGGATTGTTGATAGCCTGGAACGTCTGGGTATCCATCGCCACTTCAAAGATGAAATTAAATCAGCCCTGGACTATGTGTACTCGTATTGGGGCGAAAACGGCATTGGTTGCGGCCGTGAAAGCGTGGTTACCGATCTGAACAGCACGGCACTGGGTCTGCGTACCCTGCGTCTGCATGGCTATCCGGTTAGCTCTGATGTCTTTAAAGCCTTCAAGGGTCAGAACGGCCAATTTTCATGTTCGGAAAATATCCAGACCGATGAAGAAATTCGTGGTGTGCTGAACCTGTTTCGCGCGTCCCTGATTGCCTTCCCGGGCGAAAAAATCATGGATGAAGCAGAAATTTTCTCAACCAAATACCTGAAAGAAGCTCTGCAAAAAATCCCTGTCAGTTCCCTGAGCCGCGAAATTGGTGATGTGCTGGAATATGGCTGGCACACGTACCTGCCGCGTCTGGAAGCCCGCAACTATATTCAGGTGTTTGGTCAAGATACCGAAAACACGAAAAGCTACGTTAAATCTAAAAAACTGCTGGAACTGGCAAAACTGGAATTTAATATCTTCCAGAGTCTGCAAAAACGTGAACTGGAATCCCTGGTTCGCTGGTGGAAAGAATCAGGCTTTCCGGAAATGACCTTCTGCCGTCATCGCCACGTCGAATATTACACGCTGGCATCTTGTATTGCTTTTGAACCGCAGCATAGTGGTTTTCGTCTGGGCTTCGCGAAAACCTGCCACCTGATCACGGTTCTGGATGACATGTATGATACCTTTGGTACGGTGGATGAACTGGAACTGTTCACCGCAACGATGAAACGTTGGGACCCGAGCAGCATCGACTGTCTGCC |

Reference

Hussain, M. H., Hong, Q., Zaman, W. Q., Mohsin, A., Wei, Y., Hang, H., Zhuang, Y., Guo, M. (2021). Rationally optimized generation of integrated Escherichia coli with stable and high yield lycopene biosynthesis from heterologous mevalonate (MVA) and lycopene expression pathways. Synth. Syst. Biotechnol. 6, 85-94. doi: 10.1016/j.synbio.2021.04.001.
